# Virulence factors and biofilm forming ability of *Staphyloccoccus* species isolated from skeletal lesions of broiler chickens

**DOI:** 10.1038/s41598-025-95006-w

**Published:** 2025-03-28

**Authors:** Gustaw M. Szafraniec, Dorota Chrobak-Chmiel, Izabella Dolka, Krzysztof Adamczyk, Kamil Sułecki, Beata Dolka

**Affiliations:** 1 Department of Pathology and Veterinary Diagnostics, Institute of Veterinary Medicine, Warsaw University of Life Sciences – SGGW, Nowoursynowska 159c St, Warsaw, 02-776 Poland; 2 Department of Preclinical Sciences, Institute of Veterinary Medicine, Warsaw University of Life Sciences – SGGW, Nowoursynowska 159c St, Warsaw, 02-776 Poland; 3 Warsaw, Poland

**Keywords:** Chicken, Lameness, BCO, Staphylococcus, Arthritis, Osteomyelitis, Biofilm, Pathogens, Infectious-disease epidemiology

## Abstract

Lameness in poultry is a significant issue in modern meat production that adversely affects both animal welfare and economic outcomes due to poor leg health, reduced locomotor function, increased feed conversion ratios, and poor performance. Fast-growing broilers are particularly susceptible to lameness, with *Staphylococcus* being a major bacterial cause of skeletal infections. The aim of this study was to identify *Staphylococcus* species isolated from skeletal lesions in broiler chickens and to characterize the factors that facilitate such infections. Bacterial strains were isolated from 25 commercial broiler flocks in eastern Poland. The median prevalence of *Staphylococcus* in birds per flock was 60%. In total, 47% of the examined chickens and 88% of the examined flocks tested positive for *Staphylococcus.* The main bone sites affected by staphylococci were the femur (56.7%), femoral head (necrosis) (34.3%), hock joints (9.0%), femoral head (transient necrosis) (9.0%), tibiotarsus (7.5%), foot pads (dermatitis) (3.0%), and stifle (knee) joints (1.5%). Of all 93 *Staphylococcus* strains, 59% (55/93) were isolated from the femora. Twelve staphylococcal species were identified, all coagulase-negative, where *Staphylococcus cohnii* (24.7%) was the most prevalent species, followed by *S. epidermidis* (16.1%), *S. hominis* (15.1%), *S. lentus* (10.8%), *S. saprophyticus* (9.7%), *S. chromogenes* (8.6%), *S. arlettae* (4.3%), *S. sciuri* (4.3%), *S. haemolyticus* (2.2%), *S. xylosus* (2.2%), *S. carnosus* (1.1%), and *S. gallinarum* (1.1%). Eleven and six different staphylococcal species were implicated in the pathogenesis of femoral and tibiotarsal lesions, respectively. More than one *Staphylococcus* species was isolated from 47.8% of all *Staphylococcus*-positive chickens. Nearly all (97.8%) of coagulase-negative staphylococci isolates had biofilm-forming ability, but most of them were categorized as weak biofilm producers. The highest biofilm production was observed in the strains that caused femoral head osteonecrosis and footpad dermatitis. *Staphylococcus chromogenes*,* S. lentus*, and *S. epidermidis* exhibited the highest DNase and/or gelatinase activity. Despite the low prevalence of certain adhesin genes, the *eno* gene encoding laminin-binding protein was highly represented in staphylococci (75.3%). The study highlights the complex nature of coagulase-negative staphylococcal infections in poultry and underscores the need for further research into their virulence mechanisms and control strategies.

**Supplementary Information:**

The online version contains supplementary material available at 10.1038/s41598-025-95006-w.

## Introduction

Lameness is a prevalent and critical problem in modern poultry production, leading to impaired locomotion and reduced mobility^1,2^. This condition not only adversely affects animal welfare, but also results in significant economic losses due to increased feed conversion ratios (FCR), decreased production, higher mortality and culling rates of the affected birds^3–7^. The etiology of lameness encompasses genetic, environmental, nutritional, and infectious factors. Fast-growing broiler chickens (meat-type) are particularly susceptible to lameness because their skeletons and bone strength cannot keep up with rapid early muscle growth and body weight gain^8–10^. Most skeletal infections in poultry are caused by *Staphylococcus* spp. bacteria, and *Enterococcus* spp. and *Escherichia coli*are also frequently identified in bone lesions^2,11–13^. Bacteria of the genus *Staphylococcus*are ubiquitous in the environment (air, water, dust, soil), and they are among the most frequently isolated opportunistic bacteria that colonize the skin and mucous membranes in humans and animals, including poultry^14–16^. Staphylococci can cause a wide range of clinical infections in poultry, collectively referred to as staphylococcosis. Local and chronic infections of the skeletal system are most common^17^. Typically, the disease manifests when the host’s innate immune defenses are compromised^1,11,18,19^.

Depending on their ability to produce coagulase, staphylococci can be classified as coagulase-positive, coagulase-variable, or coagulase-negative. Coagulase-positive staphylococci (CoPS), such as *S. aureus*, are most pathogenic and are associated with the greatest health risks. In turn, coagulase-negative staphylococci (CoNS) are generally regarded as commensal and less pathogenic bacteria, and disease outbreaks caused by these strains are less frequently reported^20–23^. In recent years, CoNS have been increasingly recognized as potential pathogens, especially as etiological factors of chronic infections in both humans and poultry^24–30^.

The pathogenicity of staphylococci is associated with the expression of virulence factors, including toxins and immune-modulatory factors. The virulence factors linked with CoNS have been rarely described in skeletal infections in poultry. One of the virulence factors that enables these bacteria to infect the host organism is their ability to produce various adhesins. Adhesins are molecular structures that are found on the surface of bacteria and enable pathogens to attach to host cells and tissues. Adhesins play a critical role in the initial stages of infection by binding to specific receptor molecules on the surface of host cells, which facilitates pathogen attachment and colonization. This mechanism is a crucial step in the infectious process, allowing the pathogen to resist physical removal and evade the host’s immune defenses. Furthermore, by mediating the attachment and aggregation of bacteria, adhesins are directly implicated in biofilm formation^31,32^. Bacterial biofilms are structured communities of bacteria that adhere to a surface and become embedded in a self-produced matrix of extracellular polymeric substances (EPS). Biofilms provide a protective environment for bacteria, enhancing their resistance to antibiotics and the host immune system, and they facilitate chronic infections^33,34^.

In young chickens, the distinct anatomical and physiological characteristics of the distal ends of long bones, such as the structure of the growth plate (physis), slow blood flow, and the specific arrangement and structure of blood vessels, predispose these regions to colonization by blood-borne bacteria^35–37^. In chickens, the growth plate is more susceptible to mechanical damage due to the rapid growth rate of poultry and the associated excessive biomechanical forces. Damage to the physis can cause local ischemic necrosis or the development of sequestra that promote bacterial colonization beyond the reach of the host’s immune system^9^. These sites can eventually become infected and necrotized, leading to lameness. This condition is called bacterial chondronecrosis with osteomyelitis (BCO), and it is one of the leading causes of lameness in poultry. Staphylococci, enterococci, *E. coli*, or salmonellae can be implicated in BCO^2,9^.

The aim of this study was to identify *Staphylococcus* species isolated from skeletal lesions in broiler chickens and to characterize the cell-dependent factors that facilitate such infections in poultry.

## Materials and methods

### Sample collection

Bacterial strains were isolated from 25 commercial flocks of broiler chickens reared mostly in various regions of eastern Poland. Within 24 h post-mortem, chicken carcasses were transported to the Department of Pathology and Veterinary Diagnostics, Institute of Veterinary Medicine of the Warsaw University of Life Sciences – SGGW, for necropsy by on-farm veterinarians. All tissue samples and bacterial strains were obtained post-mortem from chickens that had died at the farm due to disease or had been euthanized at the farm by veterinarians or trained farm personnel due to poor health and welfare (lameness), in accordance with standard agricultural practices. During necropsy, special attention was paid to the extent of bone and joint lesions. Sterile cotton swabs were used to collect samples from lesions. Additionally, choanal cleft swabs and samples of bone marrow were collected to identify other infectious factors. Selected bones were sampled for histopathological examination and fixed in 10% formalin. The severity of footpad lesions (footpad dermatitis – FPD) was assessed on a scale of 0–2^3^.

## Bone marrow and synovial fluid sampling

The carcasses were rinsed before any cutting or sample collection to minimize the risk of surface contamination by airborne particulates. Prior to bone assessment, the skin covering the pelvic limbs was removed to reduce external contamination. The bones were then freed from the surrounding soft tissues, and their external surfaces were disinfected with 70% alcohol and allowed to air-dry. The epiphyses were aseptically transected with a sterile surgical blade. Immediately after transection, bone marrow was collected from the exposed site with a sterile swab. The skin surrounding the joint was disinfected with 70% alcohol before synovial fluid sampling. A sterile blade was then used to incise the joint capsule, and synovial fluid was sampled directly from the joint space with a sterile swab. The swabs were promptly transported to a laboratory, where they were streaked onto sterile microbiological media. All procedures were conducted under aseptic conditions to prevent cross-contamination and to ensure the reliability of the obtained bacterial isolates.

This study does not fall under the scope of regulations or ethical requirements for research involving live vertebrates. According to the Polish Act of 15 January 2015 on the Protection of Animals Used for Scientific or Educational Purposes (Journal of Laws of 2015, item 266 with further amendments), which implements Directive 2010/63/EU, the definition of a “procedure” explicitly excludes the killing of animals solely for the use of their organs or tissues. This study involved only post-mortem collection of tissues; therefore, it did not meet the legal definition of a “procedure” and did not require the approval of an ethics committee. All protocols and procedures were performed in accordance with the relevant guidelines/regulations of the Institute of Veterinary Medicine, Warsaw University of Life Sciences – SGGW.

## Bacterial cultures

Immediately after necropsy, the swabs from skeletal lesions were streaked onto Columbia Agar plates supplemented with 5% sheep blood (GRASO Biotech, Poland). Other media, including MacConkey Agar, Enterococcosel Agar, Mueller-Hinton Agar, and Mannitol Salt Agar (GRASO Biotech, Poland), were also used to differentiate the cultured bacteria. The plates were incubated at 37 °C for 24 h under aerobic conditions (excluding Enterococcosel agar which was incubated under microaerophilic conditions). After incubation, colonies exhibiting growth patterns and morphology characteristic of *Staphylococcus* species, such as those observed on Columbia agar – white, grey, or golden-yellow pigmentation, round, smooth, and opaque appearance, were selected for further analysis.

## Identification of Staphylococci

Selected colonies presumptively identified as staphylococci were subjected to a series of confirmatory tests.

*Gram staining and microscopy*: the presence of purple-colored cocci, occurring alone, in pairs or in clusters, confirmed the presence of Gram-positive bacteria and pathogens of the genus *Staphylococcus*.

*Gram staining and microscopy*: the presence of purple-colored cocci, occurring alone, in pairs or in clusters, confirmed the presence of Gram-positive bacteria and pathogens of the genus *Staphylococcus*.

*Catalase test*: the production of oxygen bubbles after the application of a drop of hydrogen peroxide indicated a positive catalase reaction characteristic of the genus *Staphylococcus*.

*Oxidase test*: the absence of changes in the color of bacterial colonies in the oxidase test strip (GRASO Biotech, Poland) confirmed the lack of cytochrome c oxidase, which helped differentiate staphylococci from most *Micrococcus* species.

*Coagulase test*: all isolates were tested for the production of two forms of coagulase: free (extracellular) coagulase in the tube coagulase test, and bound coagulase (clumping factor) in the slide coagulase test^38^. Coagulases contribute to the development of pseudocapsules that promote abscess formation, bacteremia, and chronic infections. They are involved in blood clotting, immune evasion, biofilm formation, and adhesion^39–41^.

### Molecular identification of *Staphylococcus* species

Following preliminary identification, the isolates were further analyzed to ascertain their species-level identity. DNA was extracted using the Genomic Mini AX Staphylococcus Spin kit (A&A Biotechnology, Poland) according to the manufacturer’s instructions. The 16 S rDNA gene fragment was amplified according to Al-Rubaye et al.^26^. The primers and PCR conditions for species identification are listed in Table 1. PCR products containing amplified 16 S rDNA fragments were sent to an external laboratory for Sanger sequencing to determine the nucleotide sequences of bacterial DNA. The obtained sequences were analyzed and compared against the NCBI (National Center for Biotechnology Information) database using the BLAST (Basic Local Alignment Search Tool) algorithm. *Staphylococcus* species were identified based on sequence similarity to known bacterial genomes in the database. In isolates identified as *Staphylococcus cohnii* (based on 16 S sequencing), which were most prevalent in the group of the tested samples, additional genetic markers were examined to refine the identification. Specifically, *rpoB* (RNA polymerase beta subunit) and *sodA* (superoxide dismutase A) genes were targeted for further PCR amplification and sequencing to provide additional markers that can help distinguish closely related *Staphylococcus* species.

**Table 1:** Nucleotide sequences of primers, anticipated amplicon size and PCR conditions for amplification of adhesin genes in *Staphylococcus* spp. Used in this study.

Gene name	Gene abbreviation	Primer sequence (5’ – 3’)	Amplicon size (bp)	PCR conditions	References
Intercellular adhesin A	*icaA*	F: GAGGTAAAGCCAACGCACTC R: CCTGTAACCGCACCAAGTTT	151	Initial denaturation (5 min at 94 °C); 25 cycles of amplification (denaturation for 45s at 94 °C, annealing for 45s at 50 °C, extension for 45s at 72 °C); final extension (5 min at 72 °C)	[1]
Intercellular adhesin D	*icaD*	F: ACCCAACGCTAAAATCATCG R: GCGAAAATGCCCATAGTTTC	211		
Intercellular adhesin B gene	*icaB*	ATACCGGCGACTGGGTTTAT R: TTGCAAATCGTGGGTATGTGT	140	Initial denaturation (5 min at 94 °C); 25 cycles of amplification (denaturation for 45s at 94 °C, annealing for 45s at 52 °C, extension for 45s at 72 °C); final extension (5 min at 72 °C)	
Intercellular adhesin C	*icaC*	F: CTTGGGTATTTGCACGCATT R: GCAATATCATGCCGACACCT	209		
Bone sialoprotein-binding protein	*bbp*	F: AACTACATCTAGTACTCAACAACAG R: ATGTGCTTGAATAACACCATCATCT	575	Initial denaturation (5 min at 94 °C); 25 cycles of amplification (denaturation for 1 min at 94 °C, annealing for 1 min at 55 °C, extension for 1 min at 72 °C); final extension (10 min at 72 °C)	[2]
Collagen adhesin	*cna*	F: GTCAAGCAGTTATTAACACCAGAC R: AATCAGTAATTGCACTTTGTCCACTG	423		
Laminin-binding protein	*eno*	F: ACGTGCAGCAGCTGACT R: CAACAGCATYCTTCAGTACCTTC	302		
Elastin-binding protein	*ebpS*	F: CATCCAGAACCAATCGAAGAC R: CTTAACAGTTACATCATCATGTTTATCTTTG	186		
Fibronectin-binding protein A	*fnbA*	F: GTGAAGTTTTAGAAGGTGGAAAGATTAG R: GCTCTTGTAAGACCATTTTTCTTCAC	643		
Fibronectin-binding protein B	*fnbB*	F: GTAACAGCTAATGGTCGAATTGATACT R: CAAGTTCGATAGGAGTACTATGTTC	524		
Fibrinogen-binding protein	*fib*	F: CTACAACTACAATTGCCGTCAACAG R: GCTCTTGTAAGACCATTTTCTTCAC	404		
Biofilm-associated protein	*bap*	F: CCCTATATCGAAGGTGTAGAATTGCAC R: GCTGTTGAAGTTAATACTGTACCTGC	971	Initial denaturation (2 min at 94 °C); 40 cycles of amplification (denaturation for 20s at 94 °C, annealing for 20s at 42 °C, extension for 50s at 72 °C); final extension (5 min at 72 °C)	[3]

1. Nourbakhsh, F. & Namvar, A. E. Detection of genes involved in biofilm formation in Staphylococcus aureus isolates. *GMS Hyg. Infect. Control* **11**, Doc07 (2016).

2. Tristan, A. et al. Use of multiplex PCR to identify Staphylococcus aureus adhesins involved in human hematogenous infections. *J. Clin. Microbiol.* **41**, 4465–4467 (2003).

3. Cucarella, C. et al. Role of Biofilm-Associated Protein Bap in the Pathogenesis of Bovine Staphylococcus aureus. *Infect. Immun.* **72**, 2177–2185 (2004).

### Phenotypic characterization of *Staphylococcus* isolates

After genetic identification of *Staphylococcus* isolates, their phenotypic characteristics were further assessed through a series of enzymatic and biochemical tests. The following tests were performed in addition to coagulase, catalase, and oxidase tests:

*DNase test*: This assay evaluates bacteria’s ability to produce deoxyribonuclease (DNase), an enzyme that hydrolyzes DNA. The bacterial isolate is incubated on a DNase agar plate (GRASO Biotech, Poland), and hydrochloric acid (HCl) is subsequently added to the medium. The presence of clear halos around the colonies is indicative of DNase activity and confirms the production of the enzyme.

*Gelatinase test*: The production of gelatinase was assessed by culturing the isolates on gelatin agar (BD, USA). Microbial ability to liquefy gelatin in the medium is indicative of positive gelatinase activity.

*Biochemical tests*: Selected strains were additionally characterized using the API Staph kit (bioMérieux, France), which contains a battery of biochemical tests for the rapid identification of staphylococci. This colorimetric sensing platform tests for a broad range of enzymatic activities and provides a detailed profile of phenotypic characteristics. Bacteria were tested for their ability to ferment carbohydrates (D-glucose, D-fructose, D-mannose, D-maltose, D-lactose, D-trehalose, D-mannitol, xylitol, D-melibiose, D-raffinose, D-xylose, D-saccharose, methyl-α-D-glucopyronidase, and N-acetyl-glucosamine) and produce alkaline phosphatase, acetyl-methyl-carbinol, arginine dihydrolase, and urease.

*Biofilm production*: All isolates were also tested for biofilm production with the microtiter plate (MP) method^42–44^ with some modifications. In the present study, Brain Heart Infusion (BHI) Broth (BD, USA) was supplemented with glucose (0.25% v/v) (D-(+)-Glucose Solution (45%), Sigma-Aldrich, Poland). The isolates were incubated in BHI for 24 h at 37 °C and then diluted to a turbidity of 0.5 MacFarland. Subsequently, 200 µL aliquots of the prepared solutions of each isolate were transported to sterile flat-bottomed 96-well polystyrene plates. Each isolate was placed in a row of 8 wells, leaving one row for the negative control containing only BHI. The plates were incubated for 24 h at 37 °C. The contents of each well were removed, and the plates were washed three times with phosphate-buffered saline (PBS). The attached bacteria were heat-fixed at 60 °C for 60 min. The wells were stained with crystal violet (200 µL, 0.1% v/v) at room temperature for 15 min. The stain was removed via aspiration, and the excess was washed by placing the plate under running tap water. The plates were air-dried at room temperature for 30 min under a laminar flow hood. Bound dye was solubilized by adding 160 µL of 95% ethanol for 30 min at room temperature. The optical density (OD) of the obtained solutions was measured at a wavelength of 570 nm. Based on the OD values, the strains were classified as non-biofilm producers (OD ≤ OD_C_), weak (OD_C_ < OD ≤ 2 × OD_C_), moderate (2 × OD_C_ < OD ≤ 4 × OD_C_), or strong biofilm producers (4 × OD_C_ < OD). The cut-off OD value (ODc) was defined as three standard deviations above the mean OD of the negative control (OD_C_ = OD_avg_. negative control + 3 × SD negative control).

### Assessment of virulence factor genes in *Staphylococcus* isolates

*Staphylococcus* isolates were screened for genes encoding several virulence factors, in particular adhesins, using the PCR method. PCR Mix Plus Green (A&A Biotechnology, Poland) was used in all reactions. Genes encoding polysaccharide intercellular adhesin (*icaACBD*), fibronectin-binding proteins (*fnbA*, *fnbB*), biofilm-associated protein (*bap*), laminin-binding surface protein enolase (*eno*), extracellular matrix-binding protein (*ebpS*), fibrinogen-binding protein (*fib*), bone sialoprotein-binding protein (*bbp*), and collagen-binding protein (*cna*) were targeted. To confirm the presence of the amplified genes, the products were visualized by horizontal agarose gel electrophoresis. The PCR conditions for each analyzed gene, including primer sequences, annealing temperatures, and amplification cycles, are detailed in Table 2.

**Table 2:** Nucleotide sequences, anticipated amplicon size and PCR conditions other oligonucleotide primers used for identification of Staphylococci at the genus and species level and detection of *Mycoplasma* and avian reovirus (ARV).

Gene	Primer sequence (5’−3’)	Amplicon size (bp)	PCR conditions	References
Prokaryotic universal *16 S rRNA*	F: AGAGTTTGATCCTGGCTCAG R: GTGCGGGCCCCCGTCAATTC	928	Initial denaturation (30 s at 90 °C); 30 cycles of amplification (denaturation for 30s at 90 °C, annealing for 15s at 55 °C, extension for 1 min at 72 °C); final extension (3 min at 72 °C)	[1]
*rpoB*	F: CAATTCATGGACCAAGC R: CCGTCCCAAGTCATGAAAC	899	Initial denaturation (5 min at 94 °C); 35 cycles of amplification (denaturation for 45s at 94 °C, annealing for 1 min at 52 °C, extension for 90s at 72 °C); final extension (10 min at 72 °C)	[2]
*sodA*	F: CCITAYICITAYGAYGCIYTIGARCC R: ARRTARTAIGCRTGYTCCCAIACRTC	480	Initial denaturation (3 min at 94 °C); 30 cycles of amplification (denaturation for 30s at 94 °C, annealing for 1 min at 37 °C, extension for 45s at 72 °C); final extension (7 min at 72 °C)	[3]
The mycoplasmal *16 S rRNA* gene	F: GCTGGCTGTGTGCCTAATACA R: TGCACCATCTGTCACTCTGTTAACCTC	1013	Initial denaturation (4 min at 94 °C); 35 cycles of amplification (denaturation for 30s at 94 °C, annealing for 30s at 56 °C, extension for 30s at 72 °C); final extension (10 min at 72 °C)	[4]
*S2* gene of ARV	F: GTGCGTGTTGGAGTTTC R: ACAAAGCCAGCCATRAT	437	Reverse transcription (30 min at 50 °C); Initial denaturation (5 min at 94 °C); 35 cycles of amplification (denaturation for 30s at 94 °C, annealing for 30s at 54 °C, extension for 1 min at 72 °C); final extension (10 min at 72 °C)	[5]

1. Al-Rubaye, A. A. K. et al. Genome Analysis of Staphylococcus agnetis, an Agent of Lameness in Broiler Chickens. *PLOS ONE* **10**, e0143336 (2015).

2. Mellmann, A. et al. Sequencing and Staphylococci Identification. *Emerg. Infect. Dis.* **12**, 333–336 (2006).

3. Poyart, C., Quesne, G., Boumaila, C. & Trieu-Cuot, P. Rapid and accurate species-level identification of coagulase-negative staphylococci by using the sodA gene as a target. *J. Clin. Microbiol.* **39**, 4296–4301 (2001).

4. Lierz, M. et al. Prevalence of mycoplasmas in eggs from birds of prey using culture and a genus-specific mycoplasma polymerase chain reaction. *Avian Pathol. J. WVPA* **36**, 145–150 (2007).

5. Bruhn, S., Bruckner, L. & Ottiger, H.-P. Application of RT-PCR for the detection of avian reovirus contamination in avian viral vaccines. *J. Virol. Methods* **123**, 179–186 (2005).

## Histopathological examination

The femur bones were fixed in 10% neutral buffered formalin and transferred to the Division of Animal Pathology, Department of Pathology and Veterinary Diagnostics, Institute of Veterinary Medicine of the Warsaw University of Life Sciences (SGGW) for histopathological examination. Bone samples were decalcified in formic acid (Shandon TBD-2 decalcifier, Thermo Fisher Scientific) for 24–72 h at room temperature. They were cut into longitudinal sections and placed in 70% ethanol before processing. The samples were dehydrated in graded ethanol and xylene baths and embedded in paraffin wax. The samples were cut into 4-µm-thick sections and stained with hematoxylin and eosin (HE). The specimens were analyzed under a BX43 light microscope (Olympus, Japan) equipped with image analysis software. The samples were imaged using a 10x/22 FN eyepiece.

### Detection of other infectious factors

*Mycoplasma* spp., especially *M. synoviae* and *M. gallisepticum*, cause respiratory, reproductive, and skeletal infections in poultry^45,46^. In poultry, lameness is often caused by mycoplasmal septic arthritis which may be misdiagnosed as BCO, which is why the studied birds were examined for this condition. For the same reason, the birds were tested for avian reoviruses (ARVs) that can also cause lameness associated with arthritis and tenosynovitis^47^.

Choanal fissure swabs collected during necropsy were analyzed for the presence of *Mycoplasma* spp. in the examined birds. DNA was isolated using Swab Kits (A&A Biotechnology, Poland) according to the manufacturer’s instructions, and it was used as a *Mycoplasma*-specific template for PCR (Table 2).

The bone marrow sampled from the femora or tibiotarsi was examined for the presence of ARVs. For this purpose, bone marrow was homogenized, and RNA was isolated from the samples using the TRIzol reagent (Thermofisher Scientific, USA) according to the protocol provided by the manufacturer. Avian reoviruses were detected in the RT-PCR assay using the OneStep RT-PCR kit (EURx, Poland) under the conditions given in Table 2. PCR results were visualized by horizontal gel electrophoresis.

### Statistical analysis

Categorical data were compared using Pearson’s chi-squared (χ^2^) test. The relationships between bacteria’s biofilm-producing ability (MP assay) and adhesion genes were examined using an ordinal regression model. The same method was used to analyze the relationships between adhesin genes and the type of femoral lesion (on the assumption that FHS, FHT, and FHN are categories of increasingly severe lesions). The strength of the correlations between ordinal data was assessed based on the values of Spearman’s rank correlation coefficient.

All statistical analyses were conducted using SciPy (v. 1.13.0) and statsmodels (v. 0.14.1) libraries in Python. Differences were considered statistically significant at *P* ≤ 0.05.

## Results

### Gross lesions

**Fig. 1:**
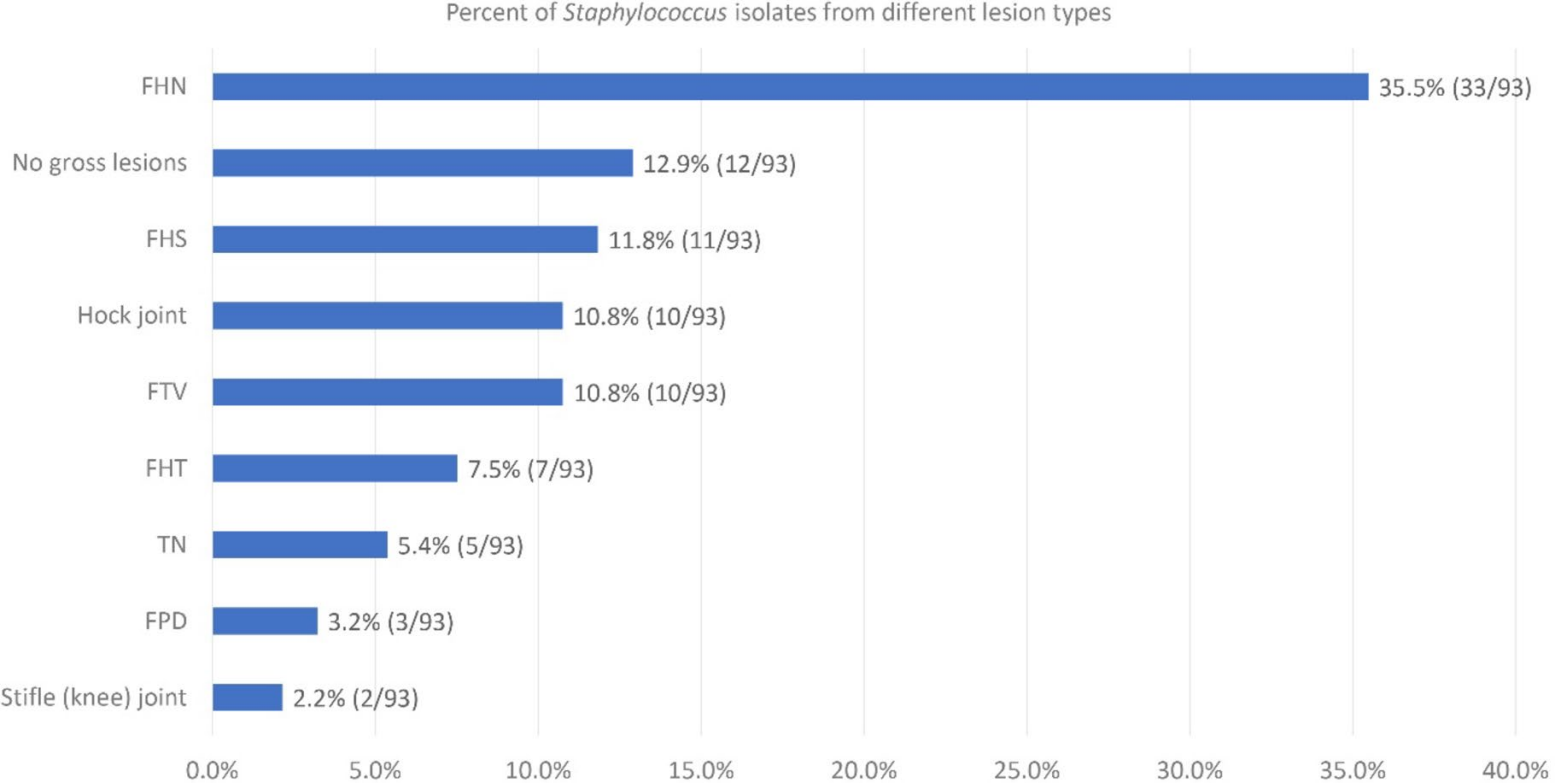
Percentage of skeletal sites from which staphylococci were isolated in broiler chickens. The values in parentheses indicate the proportion of isolates in each category to the total number of isolates (*n* = 93). FHS – femoral head separation, FHT – femoral head transitional degeneration, FHN – femoral head necrosis, TN – tibiotarsal necrosis, FTV – free thoracic vertebra, FPD – footpad dermatitis.

Depending on the severity of changes observed during macroscopic examinations, bone lesions in the proximal end of the femur can be categorized as femoral head separation (FHS), femoral head transient necrosis (FHT), and femoral head necrosis (FHN). Lesions of the proximal tibia are referred to as tibial head necrosis (THN). During necropsy, staphylococci were successfully isolated from various bone sites in chickens. FHS was identified in 7 bones, FHT was observed in 6 bones, whereas FHN, the predominant type of bone lesion, was found in 23 bones. THN was identified in 4 proximal tibiotarsal ends, and necrosis was found in one distal tibial end. Staphylococci were isolated from 2 femora and 9 tibiotarsi without gross lesions (marked as normal, N). In addition to lesions in the long bones, spinal fibriscesses in free thoracic vertebra were found in 6 birds. Staphylococci were also isolated from 6 sites of hock joint synovitis and one site of stifle (knee) joint synovitis. Staphylococcus infections associated with severe FPD were noted in 2 cases. Figure 1 presents the breakdown of isolates per lesion type.

### Identification of Staphylococci

*Staphylococcus* spp. were identified in 0–100% (mean value − 57%, median value − 60%) of the examined birds per flock. In total, 47% of the examined birds and 88% (*n* = 22) of the examined flocks tested positive for *Staphylococcus* spp. More than one *Staphylococcus* species was isolated in 47.8% (*n* = 22) of the chickens that tested positive for this pathogen.

16 S rDNA sequencing confirmed that 93 isolates belonged to the genus *Staphylococcus*. All of these strains were coagulase-negative. The most prevalent species was *Staphylococcus cohnii* that was identified in 23 isolates (24.7%). The following *Staphylococcus* species were also identified: *S. epidermidis* (*n* = 15, 16.1%), *S. hominis* (*n* = 14, 15.1%), *S. lentus* (*n* = 10, 10.8%), *S. saprophyticus* (*n* = 9, 9.7%), *S. chromogenes* (*n* = 8, 8.6%), *S. arlettae* (*n* = 4, 4.3%), *S. sciuri* (*n* = 4, 4.3%), *S. haemolyticus* (*n* = 2, 2.2%), *S. xylosus (n* = 2, 2.2%), *S. carnosus* (*n* = 1, 1.1%), and *S. gallinarum* (*n* = 1, 1.1%).

**Fig. 2:**
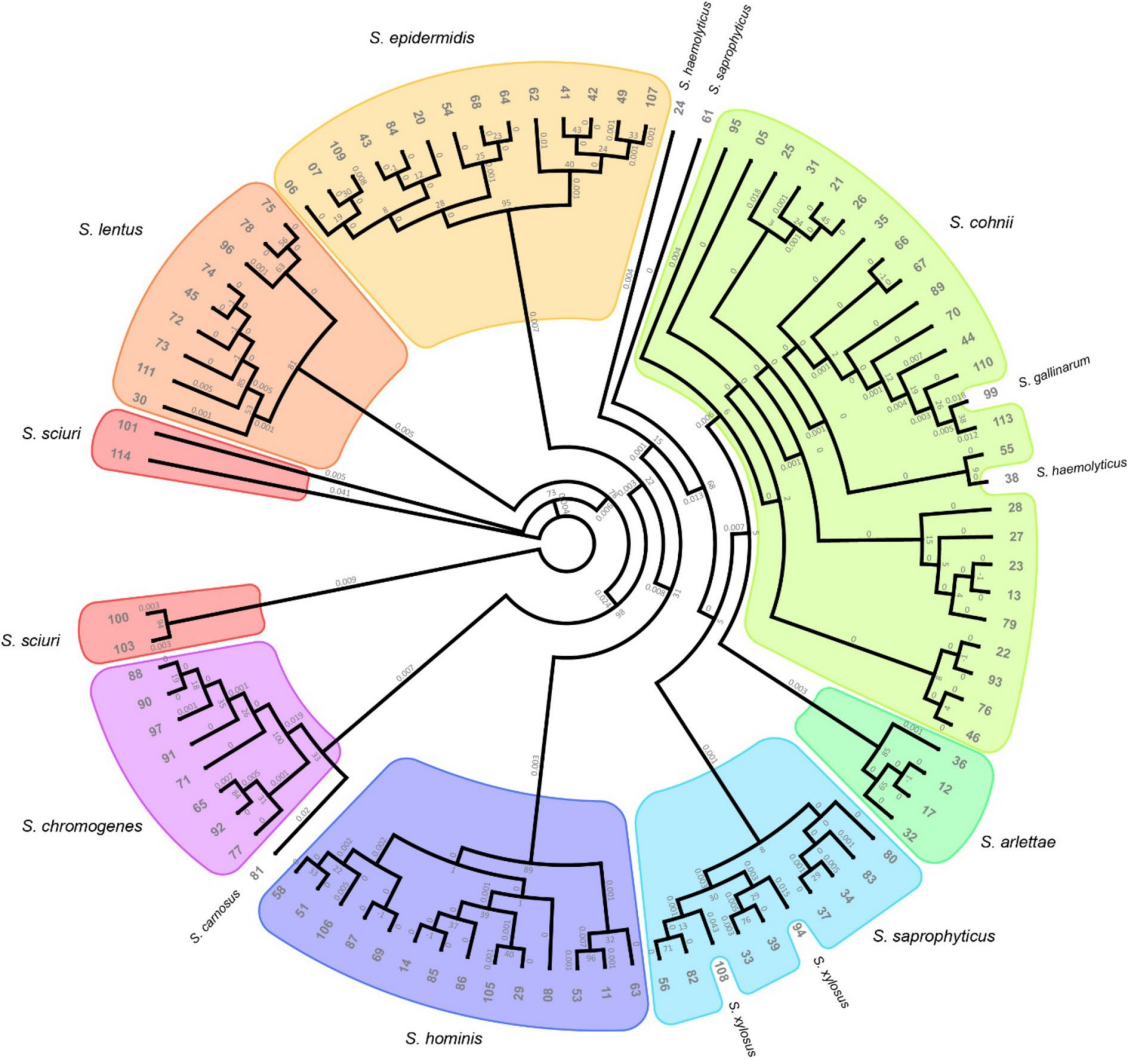
Phylogenetic tree of *Staphylococcus* strains identified based on their 16 S rDNA sequences. The tree was constructed using the maximum likelihood method with 100 bootstrap replications. Strains belonging to the same species are circled in the same color.

**Fig. 3:**
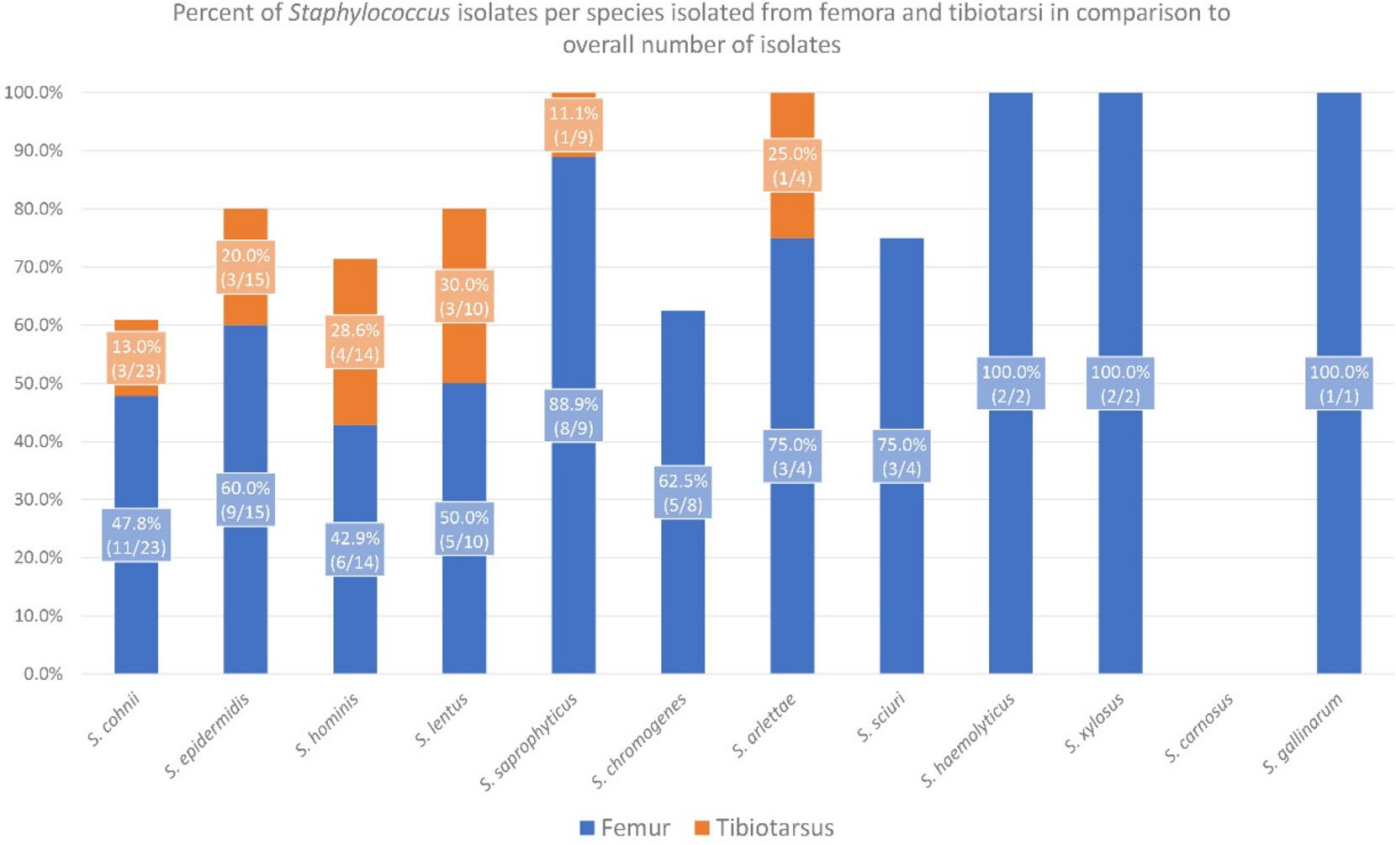
Percentage of *Staphylococcu*s species isolated from the femora and tibiotarsi in the total number of isolates. The values in parentheses indicate the proportion of isolates in each category to the total number of isolates for each species.

Madhaiyan et al.^48^ reassigned five staphylococcal species previously classified as the *Staphylococcus sciuri* group (*S. sciuri*,* S. lentus*,* S. vitulinus*, *S. stepanovicii* and *S. fleurettii*) to the genus *Mammaliicoccus*. *Staphylococcus cohnii* isolates were additionally confirmed based on the sequencing of *rpoB* and *sodA* genes. All sequences can be found in the NCBI GeneBank, and their accession numbers are provided in Supplementary Table 1. A comparison of 16 S rDNA, *rpoB*, and *sodA* sequencing results for *S. cohnii* isolates can be found in Fig. 2. The percent of *Staphylococcus* species isolated from the femora and tibiotarsi in the total number of isolates from all sites is presented in Fig. 3.

A detailed breakdown of the isolated species is presented in Table 3.

**Table 3:**
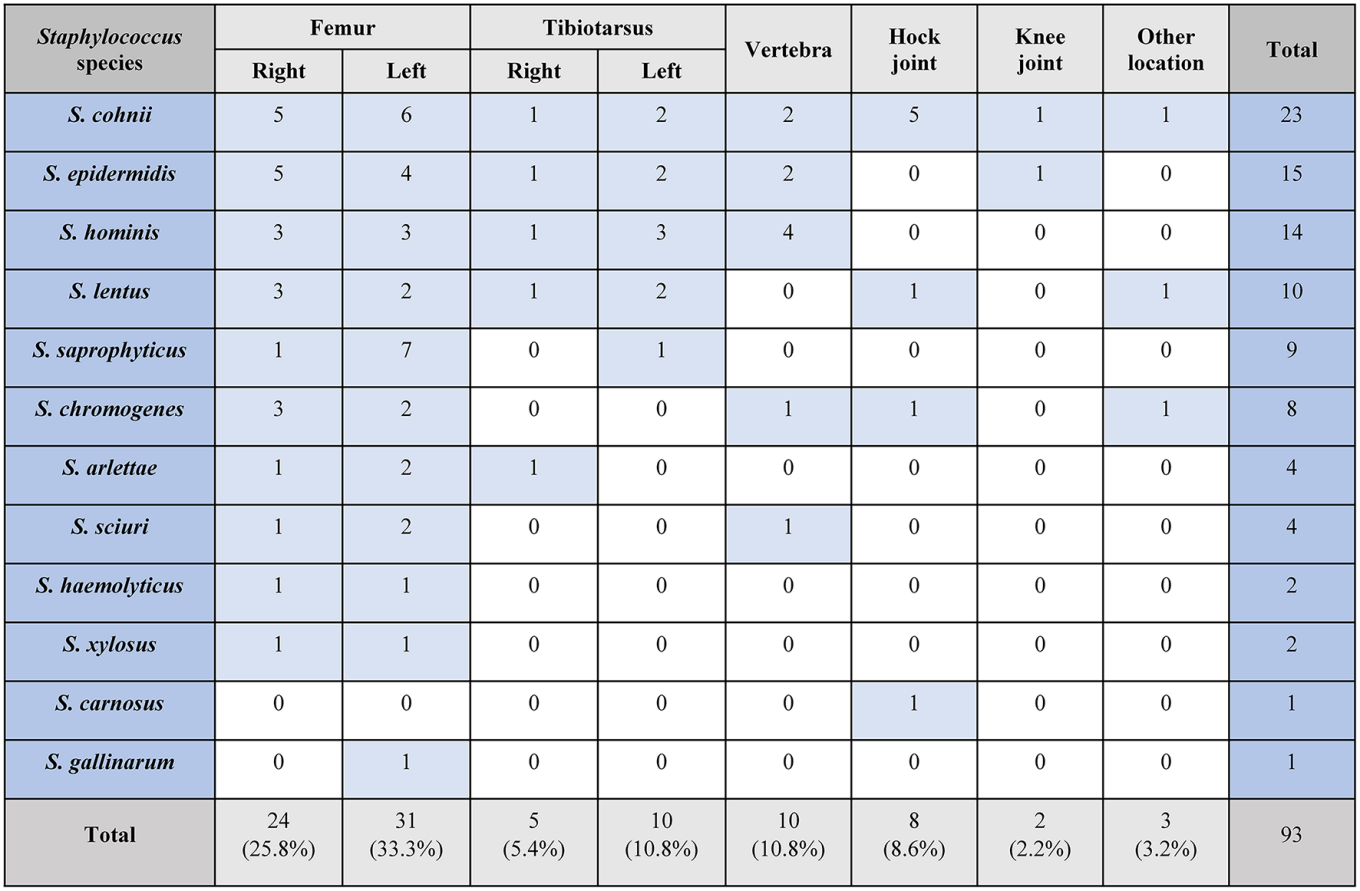
*Staphylococcus* species distribution in skeletal lesions of broiler chickens.

### Phenotypic characteristics

All isolated staphylococci were Gram-positive, catalase-positive, and oxidase-negative cocci. They did not produce free or bound coagulase. The biochemical properties of the isolates are summarized in Table 4. All *S. cohnii* isolates metabolized glucose (GLU), fructose (FRU), mannose (MNE), trehalose (TRE), and mannitol (MAN), and produced alkaline phosphatase (PAL). None of the examined *S. cohnii* isolates metabolized melibiose (MEL) or raffinose (RAF), or produced acetone (VP), glucopyranoside (MDG), and arginine (ADH). DNase was produced by 47.3% (*n* = 44) of *Staphylococcus* isolates, and gelatinase was produced by 67.7% (*n* = 63) (Table 5). Thirty-six isolates (38.7%) produced both DNase and gelatinase. Three species (excluding species with a low abundance) were the most effective producers of DNase, gelatinase, or both enzymes. All *S. chromogenes* isolates (100,0%, *n* = 8) produced both enzymes. In *S. lentus*, 70.0% (*n* = 7) of the isolates were DNase-positive, 90% (*n* = 9) were gelatinase-positive, and 70% (*n* = 7) produced both enzymes. In *S. epidermidis*, 66.7% (*n* = 10) of the isolates were DNase-positive, 80.0% (*n* = 12) were gelatinase-positive, and 60% (*n* = 9) produced both enzymes.

**Table 4:**
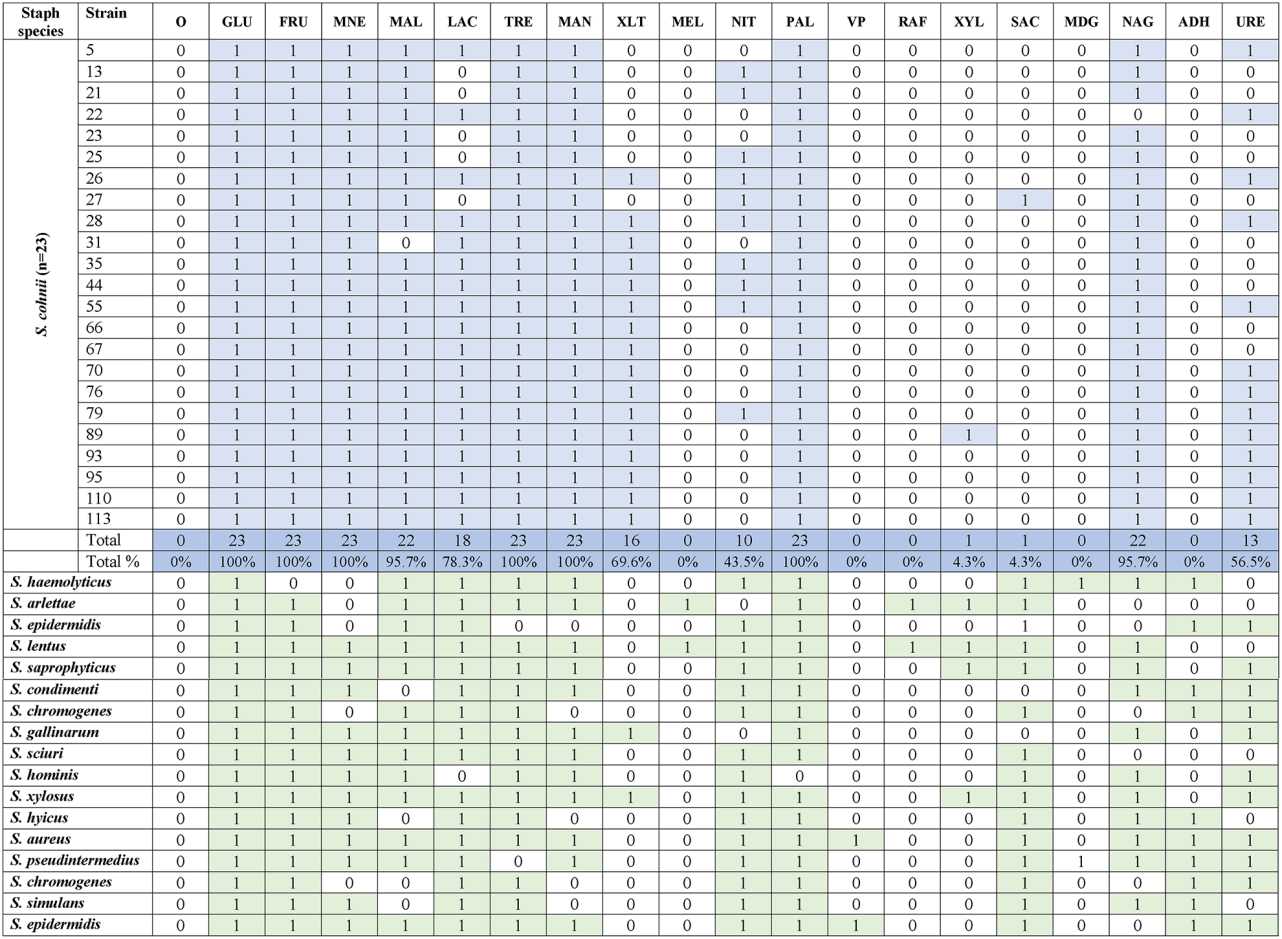
Biochemical properties as tested by API Staph strips (bioMérieux, France) for all 23 *Staphylococcus* *cohnii* and selected isolates from other *Staphylococcus* species. *S. cohnii* results are summarized.

O– no substrate, GLU – D-glucose, FRU – D-fructose, MNE – D-mannose, MAL – D-maltose, LAC – D-lactose, TRE – D-trehalose, MAN – D-mannitol, XLT– xylitol, MEL – D-melibiose, NIT – nitrates reduction, PAL – alkaline phosphatase, VP – acetyl-methyl-carbinol production, RAF – D-raffinose, XYL – D-xylose, SAC – D-saccharose, MDG – methyl-α D-glucopyranoside, NAG – N-acetyl-glucosamine, ADH – L-arginine, URE – urea.

### Biofilm-forming ability

The study demonstrated that 97.8% (91/93) of *Staphylococcus* isolates were biofilm producers. Two isolates were classified as non-biofilm producers (*n* = 2, 2.2%), whereas 65 isolates were classified as weak (*n* = 65, 69,9%), 19 isolates as moderate (*n* = 19, 20.4%), and 7 isolates as strong biofilm producers (*n* = 7, 7.5%). In most cases, strains isolated from FHN, FPD (*S. chromogenes*), and spinal fibriscesses were characterized by the highest biofilm-forming ability. The phenotypic and molecular (PCR) characteristics associated with biofilm formation and adhesin production are presented in Table 5.

**Table 5:**
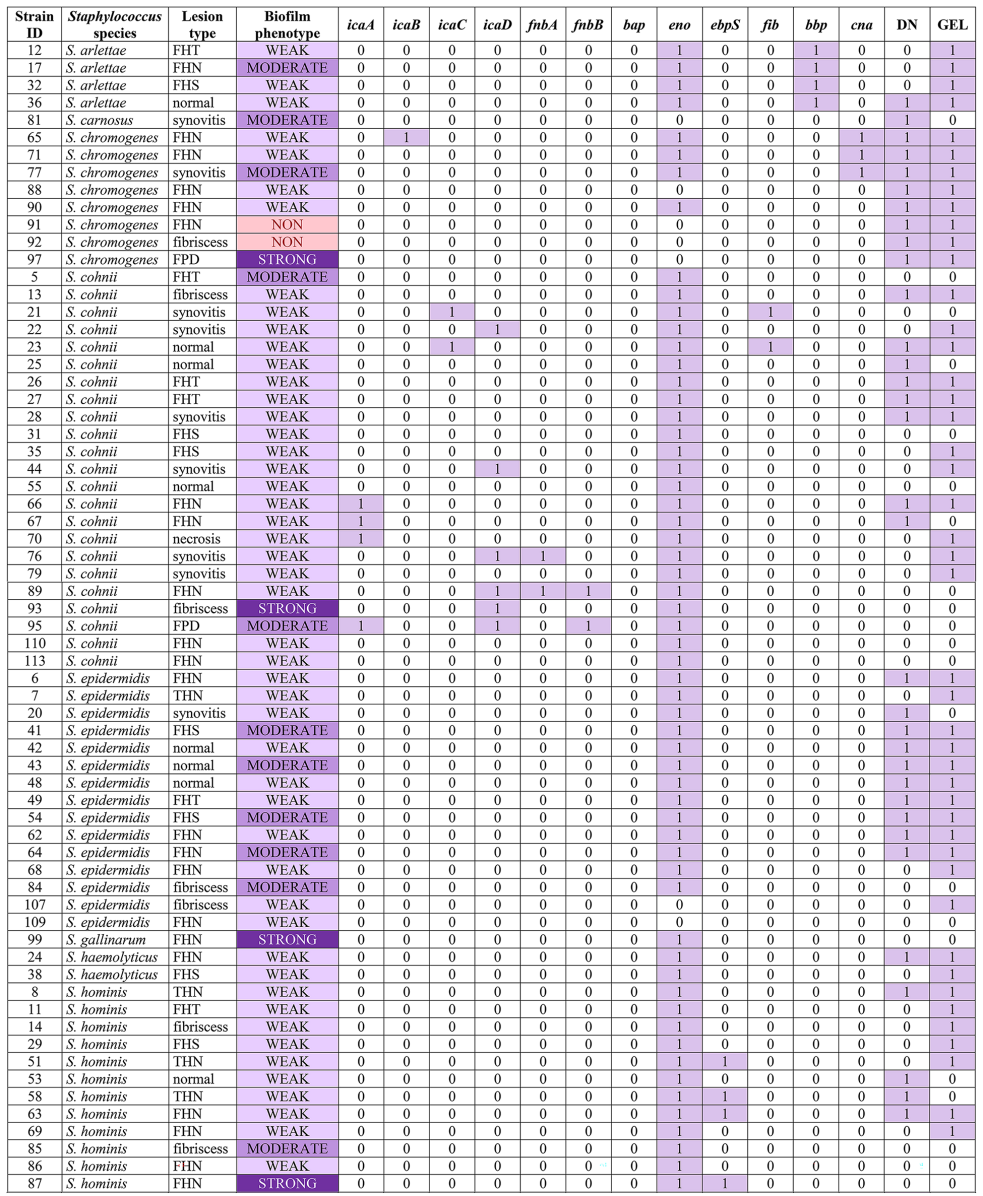

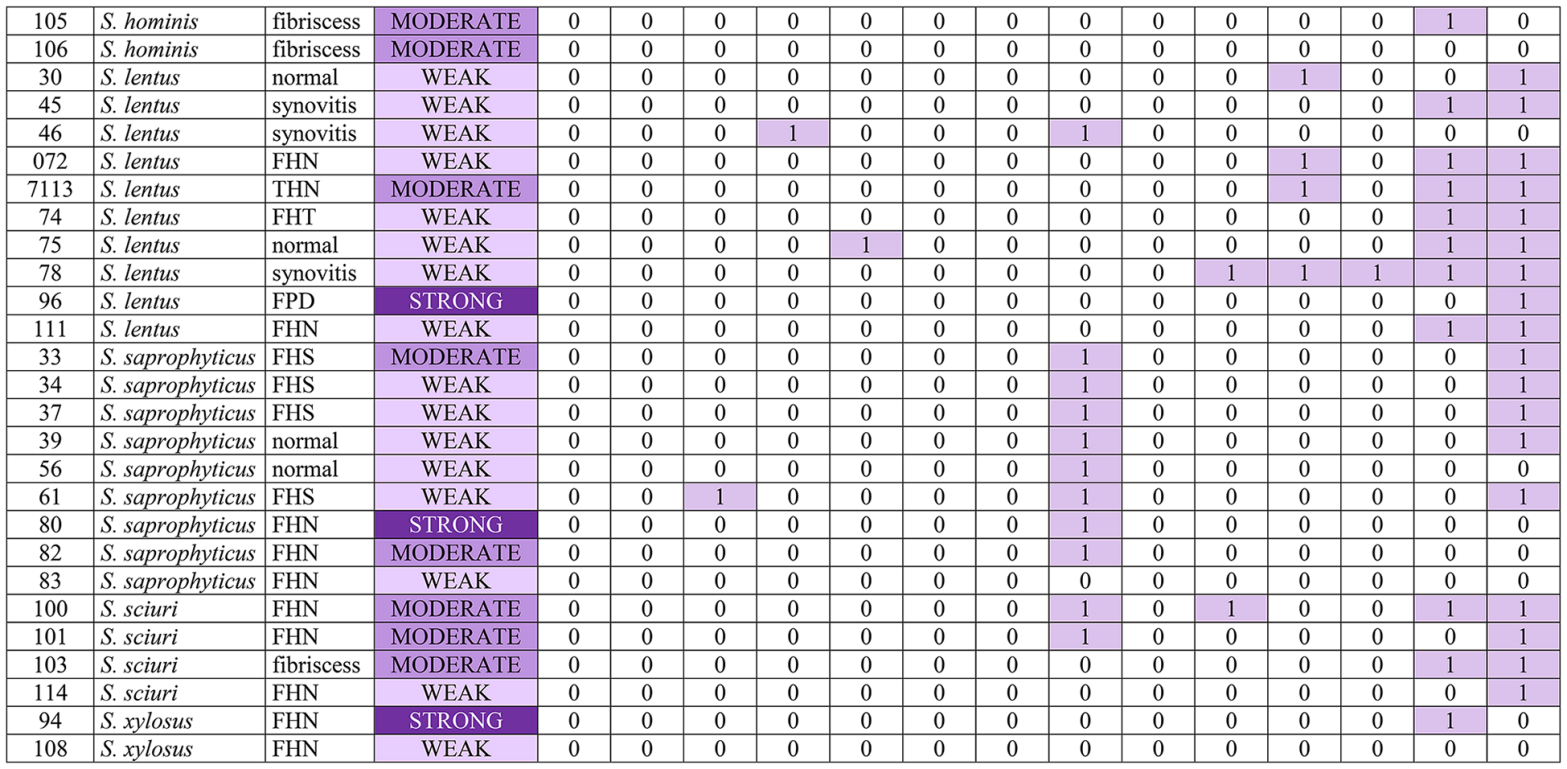
All *Staphylococcus* isolates (n=93) with type of lesion they were isolated from, biofilm phenotype assay and PCR results and adhesin genes, production of DNase and gelatinase.

icaABCD – intracellular adhesion ABCD genes, fnbAB – fibronectin-binding protein AB genes, bap – biofilm associated protein gene, eno – laminin-binding protein gene, ebpS – elastin-binding protein gene, fib – fibrinogen-binding protein gene, bbp – bone sialoprotein-binding protein gene, cna – collagen adhesin gene, DN – DNase, GEL – gelatinase, FHS – femoral head separation, FHT – femoral head transitional degeneration, FHN – femoral head necrosis, THN – tibiotarsal head necrosis.

### Molecular characteristics

In all 93 isolates tested in the PCR assay, each adhesin gene produced the following number of positive results: *icaA* *n* = 4 (4.3%), *icaB* *n* = 1 (1.1%), *icaC* *n* = 3 (3.2%), *icaD* *n* = 7 (7.5%), *fnbA* *n* = 3 (3.2%), *fnbB* *n* = 2 (2.2%), *eno* *n* = 70 (75.3%), *ebpS* *n* = 4 (4.3%), *fib* *n* = 4 (4.3%), *bbp* *n* = 8 (8.6%), *cna* *n* = 4 (4.3%). No positive results were noted for the *bap* gene. A complete breakdown of these findings can be found in Table 5. Results of Chi-Squared tests showing statistically significant (p-value < 0.05) associations between various characteristics of Staphylococci can be found in Table 6.

**Table 6:** Results of Chi-Squared tests showing statistically significant (p-value < 0.05) associations between various characteristics of Staphylococci. Results for statistically non-significant pairs can be found in supplementary materials.

Compared variables		*p*-value
Biofilm phenotype	species	0.0043
Biofilm phenotype	*eno*	0.0434
Lesion type	*icaA*	0.0001
Lesion type	*icaD*	0.0194
Lesion type	*ebpS*	0.0191
Species	*eno*	1.05e-6
Species	*ebpS*	0.0146
Species	*bbp*	3.21e-9
Species	*cna*	0.0075

*eno* – laminin-binding protein gene, *icaA* – intracellular adhesin A gene, *icaD* – intracellular adhesin D gene, *ebpS* – elastin-binding protein gene, *bbp* – bone sialoprotein binding protein gene, *cna* – collagen adhesin gene.

### Histopathology

**Fig. 4:**
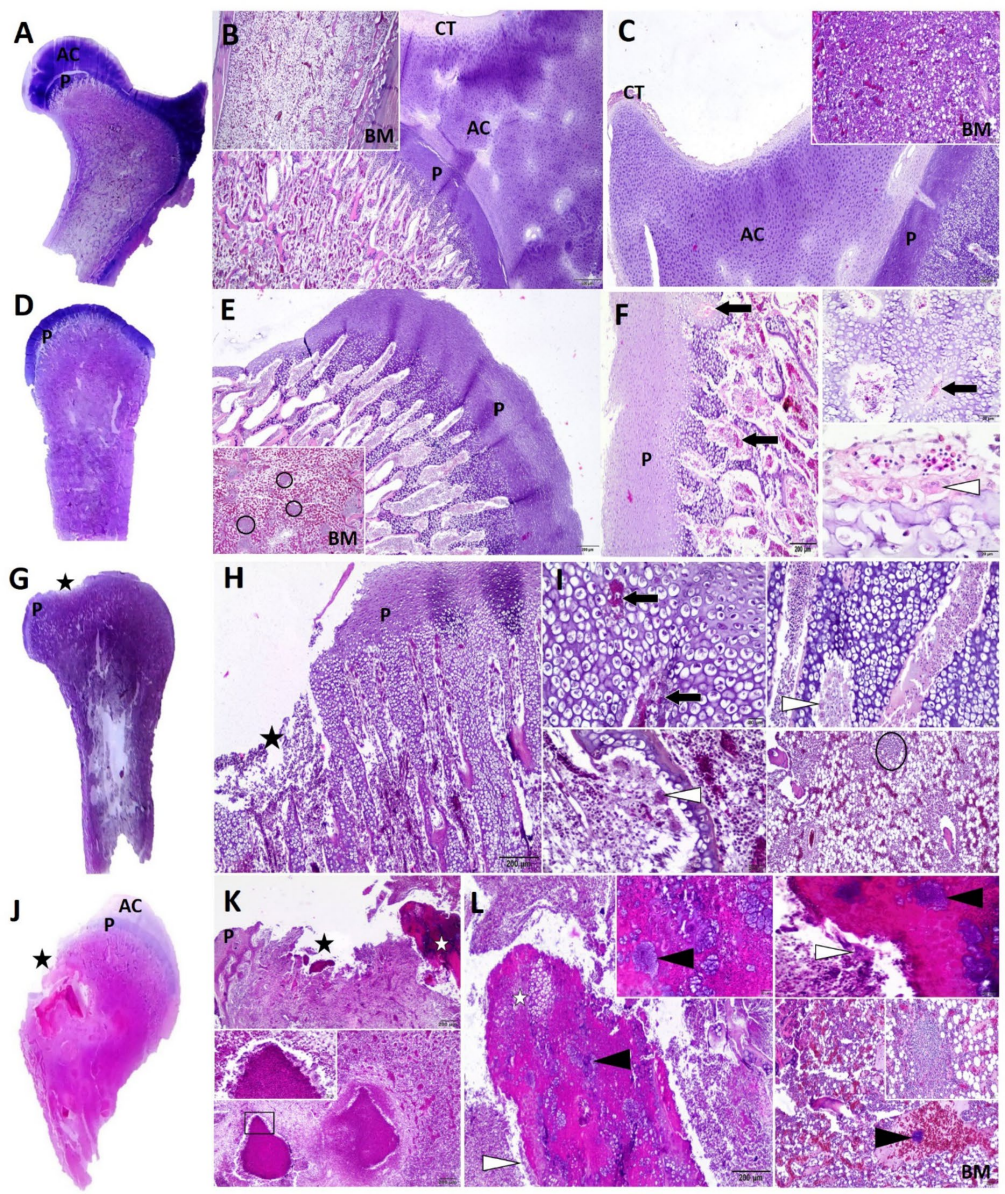
Stages of femoral head lesion in broiler chickens. A low-power cross-section of the femur bone and representative microscopic images (HE stain) of a normal femur bone (**A**–**C**), femoral head separation – FHS (**D**–**F**), femoral head transitional degeneration – FHT (**G**–**I**), femoral head necrosis – FHN (**J**–**L**). A), B) Normal femur bone, CB 42 days, and C) CB 18 days. Articular cartilage (AC) or epiphyseal cartilage (epiphysis) with a thin layer of connective tissue (CT) and the physis region (P, or growth plate/epiphyseal growth plate) beneath the epiphysis. The growth plate is complete. Bar = 200 μm, Bar = 500 μm. Inset images show the bone marrow. Bar = 500 μm, Bar = 100 μm. D), E) and F) FHS, CB 42 days. The articular cartilage (not visible) of the femoral head has been separated from the growth plate (P), revealing a rugged surface caused by irregular chondrocyte arrangement and reduced density of chondrocytes. Bar = 200 μm. The inset image in the bottom left corner shows the bone marrow (BM) with numerous lymphoid aggregates marked with black circles. Bar = 200 μm. F) Numerous dilated capillaries (neovascularization), few microthrombi (black arrows) in vascular channels. Bar = 200 μm, Bar = 20 μm. The inset image in the bottom right corner shows cartilage with reduced chondrocyte density. The cartilage matrix is lysed by multinucleated giant cells (chondroclasts, osteoclast-like cells) marked with a white arrowhead. Bar = 20 μm. G), H) and I) FHT, CB 18 days. The articular cartilage (AC) of the femoral head is separated and invisible. The loss of AC is accompanied by cartilage thinning and moderate focal defects in the physis (P), indicated by a black star. Limited fibrillation and partial fissures in the articular surface. Bar = 200 μm. Blood vessels with thrombotic occlusion (black arrow) and numerous chondroclasts (white arrowheads) in the channels can be seen in the hypertrophic zone. Bar = 20 μm. Bone marrow (BM) shows increased adipose tissue, depletion of hemopoietic stem cells, with fewer lymphoid aggregates, depletion of lymphocytes (black circle), and congestion. Bar = 100 μm. J), K) and L) FHN, CB 42 days. Marked femoral head collapse (black star) associated with disrupted bone architecture and impaired microvasculature due to the loss and necrosis of physis (P) and articular cartilage (AC). Necrosis extends from the metaphyseal-diaphyseal region deeply into the bone marrow (BM). Bar = 200 μm. Chondronecrosis is characterized by eosinophilic area (white star) with abundant bacterial colonies (black arrowheads). Necrotic cartilage is resorbed by multinucleated giant cells indicated by a white arrowhead. Bar = 20 μm. K) Images in the bottom left corner show caseous necrotic foci surrounded by multinucleated giant cells, heterophils, mononuclear cells, and fibroblasts in the bone marrow space within the diaphysis. Bar = 200 μm. The framed area in the inset in the top left corner was magnified to improve the visibility of details. Bar = 20 μm. L) The image in the bottom right corner shows osteomyelitis. Dilated blood vessels in the marrow space contain fibrin thrombi and bacterial colonies (black arrowhead). Bar = 100 μm. A vaguely bounded lymphoid aggregate is circled in the inset image. Bar = 50 μm.

Microscopic bone lesions were examined to assess the progression of BCO and the severity of damage to both the cartilage and the bone marrow cavity of the proximal femur (Fig. 4). These lesions were compared with a femur bone with a normal histology (Fig. 4A–C). Articular cartilage was not present in any stages of lesion progression (categories), beginning from FHS **(**Fig. 4D–F**)** through FHT (Fig. 4G–I**)** to FHN (Fig. 4J–L**)**, and irregular articular surfaces indicated that articular cartilage was separated from the growth plate. Damaged cartilage structure was observed in the physis and the metaphyseal region (subchondral junction), with progressive depletion of chondrocytes, disorderly arrangement of chondrocytes, and the presence of cell-free areas associated with an enlarged cartilage matrix. The morphology of chondrocytes (also known as dark chondrocytes) was indicative of apoptotic changes, displaying nuclear condensation and cell shrinkage. In addition, increased vacuolization of chondrocytes, including the presence of chondrocytes with a balloon-like appearance, single necrotic chondrocytes in lacunae, and an increased number of empty chondrocyte lacunae, were indicative of cell death. The cartilage area was thinned, revealing partial fissures and a higher number of multinucleated cartilage-resorbing cells (referred to as chondroclasts or osteoclast-like cells). In addition, numerous capillaries of varying sizes, including capillaries with microthrombi, were also observed. In the advanced stage (FHN), multiple necrotic foci were identified in the physeal and metaphyseal regions, extending into the diaphysis. They were characterized by eosinophilic areas with cellular debris containing necrotic chondrocytes (visible as cell shadows with outlines), osseous trabeculae, and inflammatory cells consisting mainly of damaged heterophils, fibrin deposits, and large intralesional cocci colonies. Necrotic areas had the appearance of caseating granulomas, with the central cellular debris surrounded by multinucleated giant cells, heterophils, lymphocytes, macrophages, and fibroblasts. Lymphoid aggregates were present in early-stage bone marrow lesions, whereas necrotic areas, bacterial thrombi, inflammatory cells, and trabecular bone resorption were observed in the final stage (FHN).

### Other infectious factors

All samples tested negative for both avian reoviruses and *Mycoplasma* spp.

## Discussion

Historically, CoNS have been considered less pathogenic than their coagulase-positive counterparts, but their pathogenic potential has been recognized based on emerging evidence^29,49^. Despite the above, many modern veterinary medicine textbooks claim that *S. aureus*, a coagulase-positive species, is the most probable cause of septic arthritis or osteomyelitis (BCO) in animals that test positive for staphylococci^50,51^. In the present study, *S. aureus* was not identified in any of the tested isolates, which was an unexpected result. All 12 *Staphylococcus* species isolated from skeletal lesions in chicken belonged to CoNS, and the most prevalent species was *S. cohnii*, followed by *S. epidermidis*, *S. hominis*, and *S. lentus*. The variability in research results could be attributed to geographic differences in species distribution, although considerable discrepancies are unlikely to occur in modern poultry supply chains. A similar study was conducted in Poland by Wieliczko et al.^52^ who isolated staphylococci from broiler chickens with various types of staphylococcosis, not only limited to the skeletal system. In the cited research, only 21.0% of *Staphylococcus* isolates from broiler chickens were classified as *S. aureus*, and the remaining isolates were classified as CoNS, with a predominance of *S. xylosus* (36.8%).

This is the first study to report that *S. cohnii* was the dominant species (*n* = 23, 24.7%) of staphylococci isolated from BCO lesions. *Staphylococcus cohnii* had been previously isolated from poultry, but it was not directly associated with skeletal system infections. *Staphylococcus cohnii* was the second most prevalent *Staphylococcus* species (*n* = 6, 13.0%) after *S. aureus* (*n*= 34, 73.9%) that was isolated from the hock joints and internal organs of broilers and broiler breeders originating from the US states of Arkansas and Missouri^53^. Aarestrup et al.^54^ examined 118 staphylococcal isolates from poultry in Denmark and found that *S. cohnii* was the fourth most frequently isolated species (*n* = 6, 5.1%), preceded by *S. xylosus* (*n* = 9, 7.6%), *S. hyicus* (*n* = 11, 9.3%), and *S. aureus* (*n* = 83, 70.3%). These isolates originated mainly from birds with arthritis and flocks with increased mortality (caused by sepsis). Wieliczko et al.^52^ analyzed 60 staphylococcus isolates from various poultry species and production systems in Poland. The isolates were obtained from various tissues and body regions. *Staphylococcus cohnii* was identified in 3 isolates (5.0%) from a broiler chicken, a turkey, and a waterfowl. The most prevalent species was *S. aureus* (*n* = 29, 48.3%), followed by *S. xylosus* (*n* = 9, 15.0%) and *S. lentus* (*n* = 5, 8.3%). A different distribution of *Staphylococcus* species in Polish poultry was reported by Marek et al.^55^. They examined 302 isolates from the hearts, livers, joints, and bone marrow of broiler chickens, broiler turkeys, laying and breeding hens, and waterfowl. *Staphylococcus cohnii* was the most frequently isolated species (*n* = 70, 23.5%), followed by *S. aureus* (*n* = 48, 15.9%) and *S. lentus* (*n* = 42, 13.9%). However, the tissues and sites from which *S. cohnii* was isolated were not described in detail in the cited study.

We hypothesized that CoNS cause less severe and more chronic infections, whereas *S. aureus* causes more pronounced outbreaks that were not included in this study. Large outbreaks with a high percentage of lame birds attract significant attention and prompt more thorough diagnostic efforts, while low-scoring or subclinical cases may go unnoticed or are not pursued as extensively. Additionally, acute diseases often produce clear and specific symptoms, while chronic infections may produce more ambiguous signs, which complicates the diagnosis process.

CoNS differ considerably in phenotypic and molecular parameters. In the current study, the vast majority of the isolates produced biofilm, but only a few were identified as strong biofilm producers. This is one of the first studies to analyze the biofilm-forming ability of poultry isolates with the use of the MP method. A previous study evaluating the biofilm-forming abilities of CoNS from poultry^44^ revealed a relatively uniform split between phenotypes with different biofilm-forming potential (each class contained 20.7–27.6% of the isolates). Other studies were not performed on poultry isolates, but most of the identified staphylococci were weak biofilm producers. Milanov et al.^56^ conducted an MTP assay of *S. aureus*isolates originating from bovine mastitis, and their results were more skewed toward the weak phenotype (weak producers – 57.1%, moderate producers – 30.0%, strong producers – 12.9%). In another study of bovine mastitis^57^, 3.3% of CoNS isolates were classified as non-producers, 47.8% as weak producers, 13.3% as moderate producers, and 35.6% as strong biofilm producers. Several factors may be responsible for the differences in isolate distribution into specific biofilm production categories. Above all, the studied isolates originated from different clinical cases of varied intensity and from different animals. Bacterial cultivation methods could also play an important role. Research has shown that different culture media affect gene expression and, consequently, phenotypic traits^42,57,58^.

PCR screening revealed that many virulence genes were present only in a small fraction of the tested isolates. The above could be explained by the fact that many identification methods were based on studies of human staphylococcal osteomyelitis, or that the applied methodologies were tailored to *S. aureus*. However, a more likely explanation is that CoNS naturally exhibit fewer virulence factors or utilize factors that were not identified in this study. Even some of the isolates that were classified as strong biofilm producers in the MP assay tested negative for all adhesin genes, which confirms the observation that not all virulence factors in CoNS have been identified.

With the exception of the *eno*gene encoding laminin-binding protein, none of the studied adhesin genes were significantly associated with biofilm-forming ability. The laminin-binding protein (enolase) enables bacterial binding to laminin, a major component of the vascular basal membrane. This protein may facilitate the spread of infection to other tissues^59^. Bacterial adherence to laminin in vascular basement membranes seems especially important in the context of BCO pathogenesis, where blood-borne bacteria penetrate blood vessels and colonize the area around the physis, where blood flow is sluggish and blood vessels have wide openings^2,9^.

The low prevalence of other adhesin genes was an unexpected result. Adhesin genes were generally more prevalent in other studies of staphylococci, but these analyses tended to focus on human patients or/and *S. aureus*rather than skeletal lesions in poultry. Tristan and al^60^ reported a much higher prevalence of *bbp*,* cna*,* eno*,* ebpS*,* fnbAB*, and *fib* genes in *S. aureus* isolated from hematogenous osteomyelitis and/or arthritis in humans. However, similarly to the current study, *eno* was the most prevalent gene (100%). Marek et al.^44^ studied some of these genes (*icaAB*, *bap*, *eno*) and the biofilm-forming ability of CoNS in poultry. They analyzed isolates from various tissues, but *eno* was the predominant molecular factor that was identified in 70.1% of the isolates, whereas *icaAB* and *bap*were detected in 6.9% and 5.7% of the isolates, respectively. These findings corroborate our results and could imply that the ability to produce enolase is widespread in CoNS populations colonizing poultry. Nemati and al^61^ screened *S. aureus* isolates from both healthy and diseased chickens (affected mostly by tenosynovitis) for many of the genes analyzed in the present study (*bap*, *bbp*, *cna*, *ebpS*, *eno*, *fib*, *fnbAB*, *icaAD*). Similarly to this study, they reported a low prevalence of *bap* (0% vs. 0% in the present study), *bbp* (0% vs. 8.6%), and *fnbB* (0% vs. 2.2%) genes, which suggests that these genes could play a minor role in the pathogenesis of *Staphylococcus* infections. The *eno* gene was also highly prevalent (100%). However, considerable differences were noted in the prevalence of *cna* (52.9% vs. 4.3% in the present study), *ebpS* (89.4% vs. 4.3%), *fib* (100% vs. 4.3%), *fnbA* (100% vs. 3.2%), *icaA* (30.6% vs. 4.3%), and *icaD* (93% vs. 7.5%) genes, which implies that these virulence factors could be specific for *S. aureus*.

The histopathological changes observed in articular cartilage were indicative of ongoing necrosis, beginning with FHS and progressing to BCO in subsequent stages. Microthrombi are implicated in the etiology of BCO, and their presence in blood vessels was not surprising. The changes associated with FHT typically present as focal defects and fissures in the physis. Necrotic lesions in chondrocytes are more advanced than in FHS. In FHN, these changes are more pronounced and involve visible loss of a fragment of the femoral head, extensive necrotic and inflammatory lesions, and visible bacteria colonies. These lesions are typical of FHN/BCO^2,9,62,63^. Staphylococci isolated from macroscopically normal femora and tibiotarsi originated from birds where contralateral bones or other sites showed signs of infection. In some cases, *Staphylococcus* strains were not isolated from macroscopic lesion sites, but bacterial growth was noted when apparently normal bones were cultured. The above suggests that staphylococci can spread throughout the body and colonize susceptible sites when favorable conditions for infection arise.

**Fig. 5:**
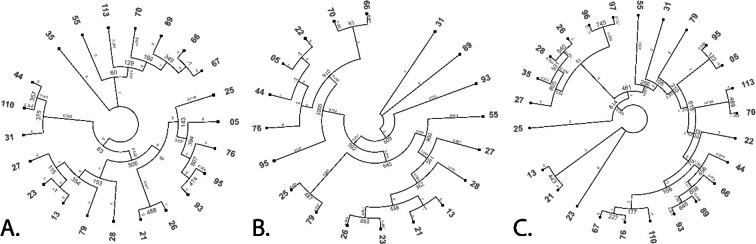
Phylogenetic trees of *Staphylococcus cohnii* strains isolated based on their 16 S rDNA (**A**), *rpoB* (**B**), and *sodA* (**C**) sequences. The trees were constructed using maximum likelihood method with 1000 bootstrap replications.

The results of 16 S rRNA sequencing effectively differentiated *Staphylococcus* species in isolates harboring several closely related species (Fig. 5). Two additional housekeeping genes, *rpoB* and *sodA*, were sequenced to confirm the identity of *S. cohnii*, the most frequently isolated species according to 16 S rDNA sequencing. Both genes supported the discrimination of *S. cohnii* isolates. 16 S rDNA appears to be the most useful gene in the tested group. It is characterized by the largest database of genetic sequences for comparative purposes and the highest percent identity (up to 100%, data not shown). Satisfactory results were also obtained for *rpoB* sequences. The percent identity of *sodA* was somewhat lower (93–97%), but *S. cohnii* still emerged as the most probable species when nBLAST was used. The phylogenetic trees generated based on the above sequences revealed differences between individual *S. cohnii* strains (Fig. 2). However, the studied strains were characterized by very small genetic differences and distances, which may complicate the analysis.

## Conclusions

This study highlights the significant role of CoNS in skeletal infections in broiler chickens, where *S. cohnii* emerged as the most prevalent species. The present findings indicate that CoNS, historically considered less pathogenic, are capable of causing chronic infections in poultry, particularly through biofilm formation. The widespread presence of the *eno* gene suggests that it plays an important role in the pathogenicity of CoNS in poultry. The absence of *S. aureus* in the examined isolates contrasts with previous findings, indicating potential regional differences or underestimation of CoNS in poultry health management. Further research is needed to explore the genetic basis of virulence in CoNS and to develop effective strategies for preventing and controlling these infections in broiler flocks.

## Electronic supplementary material

Below is the link to the electronic supplementary material.

Supplementary Material 1

## Data Availability

The data generated and analyzed in this study are included in the article (and the Supplementary Materials). All sequences can be found in the NCBI GeneBank, and their accession numbers are provided in Supplementary Table 1.

## Supplementary Information

Supporting data are presented in the article and the Supplementary Materials.

## References

[1] McNamee, P. T. & Smyth, J. A. Bacterial chondronecrosis with osteomyelitis (‘femoral head necrosis’) of broiler chickens: a review. *Avian Pathol. J. WVPA*. **29**, 253–270 (2000). 10.1080/03079450050118386 19184815

[2] Wideman, R. F. Bacterial chondronecrosis with osteomyelitis and lameness in broilers: a review. *Poult. Sci.* **95**, 325–344 (2016). 26527707 10.3382/ps/pev320

[3] Kestin, S. C., Knowles, T. G., Tinch, A. E. & Gregory, N. G. Prevalence of leg weakness in broiler chickens and its relationship with genotype. *Vet. Rec*. **131**, 190–194 (1992). 1441174 10.1136/vr.131.9.190

[4] Weeks, C. A., Danbury, T. D., Davies, H. C., Hunt, P. & Kestin, S. C. The behaviour of broiler chickens and its modification by lameness. *Appl. Anim. Behav. Sci.* **67**, 111–125 (2000). 10719194 10.1016/s0168-1591(99)00102-1

[5] Bradshaw, R. H., Kirkden, R. D. & Broom, D. A review of the aetiology and pathology of leg weakness in broilers in relation to welfare. *Avian Poult. Biol. Rev.* **13**, 45–103 (2002).

[6] Gocsik, É., Silvera, A. M., Hansson, H., Saatkamp, H. W. & Blokhuis, H. J. Exploring the economic potential of reducing broiler lameness. *Br. Poult. Sci.* **58**, 337–347 (2017). 28294637 10.1080/00071668.2017.1304530

[7] Granquist, E. G., Vasdal, G., de Jong, I. C. & Moe, R. O. Lameness and its relationship with health and production measures in broiler chickens. *Animal* **13**, 2365–2372 (2019). 30894238 10.1017/S1751731119000466 PMC6749567

[8] Thorp, B. H. & Waddington, D. Relationships between the bone pathologies, Ash and mineral content of long bones in 35-day-old broiler chickens. *Res. Vet. Sci.* **62**, 67–73 (1997). 9160428 10.1016/s0034-5288(97)90183-1

[9] Wideman, R. F. & Prisby, R. D. Bone circulatory disturbances in the development of spontaneous bacterial chondronecrosis with osteomyelitis: a translational model for the pathogenesis of femoral head necrosis. *Front. Endocrinol.* **3**, 183 (2012). 10.3389/fendo.2012.00183 PMC3550519 23346077

[10] Prisby, R. et al. Kinetic examination of femoral bone modeling in broilers. *Poult. Sci.* **93**, 1122–1129 (2014). 24795304 10.3382/ps.2013-03778

[11] Huff, G. R., Huff, W. E., Rath, N. C. & Balog, J. M. Turkey osteomyelitis complex. *Poult. Sci.* **79**, 1050–1056 (2000). 10901209 10.1093/ps/79.7.1050

[12] Dinev, I. Clinical and morphological investigations on the prevalence of lameness associated with femoral head necrosis in broilers. *Br. Poult. Sci.* **50**, 284–290 (2009). 19637027 10.1080/00071660902942783

[13] Wijesurendra, D. S. et al. Pathological and Microbiological investigations into cases of bacterial chondronecrosis and osteomyelitis in broiler poultry. *Avian Pathol. J. WVPA*. **46**, 683–694 (2017). 10.1080/03079457.2017.1349872 28669198

[14] Cooper, J. E. & Needham, J. R. An investigation into the prevalence of S aureus on avian feet. *Vet. Rec*. **98**, 172–174 (1976). 1258319 10.1136/vr.98.9.172

[15] Nagase, N. et al. Isolation and species distribution of Staphylococci from animal and human skin. *J. Vet. Med. Sci.* **64**, 245–250 (2002). 11999444 10.1292/jvms.64.245

[16] Syed, M. A. et al. Staphylococci in poultry intestines: a comparison between farmed and household chickens. *Poult. Sci.* **99**, 4549–4557 (2020). 32867999 10.1016/j.psj.2020.05.051 PMC7598113

[17] Szafraniec, G. M., Szeleszczuk, P. & Dolka, B. Review on skeletal disorders caused by Staphylococcus spp. In poultry. *Vet. Q.* **42**, 21–40 (2022). 35076352 10.1080/01652176.2022.2033880 PMC8843168

[18] Harry, E. G. Some characteristics of Staphylococcus aureus isolated from the skin and upper respiratory tract of domesticated and wild (Feral) birds. *Res. Vet. Sci.* **8**, 490–499 (1967). 5183295

[19] Wideman, R. F. & Pevzner, I. Dexamethasone triggers lameness associated with necrosis of the proximal tibial head and proximal femoral head in broilers. *Poult. Sci.* **91**, 2464–2474 (2012). 22991529 10.3382/ps.2012-02386

[20] Rich, M. Staphylococci in animals: prevalence, identification and antimicrobial susceptibility, with an emphasis on methicillin-resistant Staphylococcus aureus. *Br. J. Biomed. Sci.* **62**, 98–105 (2005). 15997888 10.1080/09674845.2005.11732694

[21] Peton, V. & Le Loir, Y. Staphylococcus aureus in veterinary medicine. *Infect. Genet. Evol.* **21**, 602–615 (2014). 23974078 10.1016/j.meegid.2013.08.011

[22] Pyzik, E. et al. Detection of antibiotic resistance and classical enterotoxin genes in coagulase -negative Staphylococci isolated from poultry in Poland. *J. Vet. Res.* **63**, 183–190 (2019). 31276057 10.2478/jvetres-2019-0023 PMC6598191

[23] González-Martín, M., Corbera, J. A., Suárez-Bonnet, A. & Tejedor-Junco, M. T. Virulence factors in coagulase-positive Staphylococci of veterinary interest other than *Staphylococcus aureus*. *Vet. Q.* **40**, 118–131 (2020). 32223696 10.1080/01652176.2020.1748253 PMC7178840

[24] Tate, C. R., Mitchell, W. C. & Miller, R. G. Staphylococcus hyicus associated with Turkey stifle joint osteomyelitis. *Avian Dis.* **37**, 905 (1993). 8257392

[25] Chénier, S. & Lallier, L. Acantholytic folliculitis and epidermitis associated with *Staphylococcus hyicus* in a line of white leghorn laying chickens. *Vet. Pathol.* **49**, 284–287 (2012). 21856871 10.1177/0300985811415705

[26] Al-Rubaye, A. A. K. et al. Genome analysis of Staphylococcus Agnetis, an agent of lameness in broiler chickens. *PLOS ONE*. **10**, e0143336 (2015). 26606420 10.1371/journal.pone.0143336 PMC4659636

[27] Tsai, S. S. et al. Joint lesions in Taiwan native colored broiler chicken with natural and experimental *Staphylococcus aureus* or *S. cohnii* infection. *Taiwan. Vet. J.* **41**, 237–244 (2015).

[28] Argemi, X., Hansmann, Y., Prola, K. & Prévost, G. Coagulase-Negative Staphylococci pathogenomics. *Int. J. Mol. Sci.* **20**, 1215 (2019). 30862021 10.3390/ijms20051215 PMC6429511

[29] Heilmann, C., Ziebuhr, W. & Becker, K. Are coagulase-negative Staphylococci virulent? *Clin. Microbiol. Infect.* **25**, 1071–1080 (2019). 30502487 10.1016/j.cmi.2018.11.012

[30] Sorour, H. K., Shalaby, A. G., Abdelmagid, M. A. & Hosny, R. A. Characterization and pathogenicity of multidrug-resistant coagulase-negative Staphylococci isolates in chickens. *Int. Microbiol.* **26**, 989–1000 (2023). 37055707 10.1007/s10123-023-00354-0 PMC10622361

[31] Clarke, S. R. & Foster, S. J. Surface Adhesins of Staphylococcus aureus. In *Advances in Microbial Physiology* Vol. 51 (ed. Poole, R. K.) 187–224 (Academic, 2006). 10.1016/S0065-2911(06)51004-5 17010697

[32] Coagulase-Negative Staphylococci and Their Role in Infection. in *Molecular Medical Microbiology* 793–810 Academic Press, (2015). 10.1016/B978-0-12-397169-2.00043-3

[33] Hathroubi, S., Mekni, M. A., Domenico, P., Nguyen, D. & Jacques, M. Biofilms: microbial shelters against antibiotics. *Microb. Drug Resist. Larchmt. N*. **23**, 147–156 (2017). 10.1089/mdr.2016.0087 27214143

[34] Ciofu, O., Moser, C., Jensen, P. Ø. & Høiby, N. Tolerance and resistance of microbial biofilms. *Nat. Rev. Microbiol.* **20**, 621–635 (2022). 35115704 10.1038/s41579-022-00682-4

[35] Howlett, C. R. The fine structure of the proximal growth plate of the avian tibia. *J. Anat.* **128**, 377–399 (1979). 438096 PMC1232943

[36] Howlett, C. R., Dickson, M. & Sheridan, A. K. The fine structure of the proximal growth plate of the avian Tibia: vascular supply. *J. Anat.* **139**, 115–132 (1984). 6469851 PMC1164451

[37] Orth, M. W. & Cook, M. E. Avian tibial dyschondroplasia: a morphological and biochemical review of the growth plate lesion and its causes. *Vet. Pathol.* **31**, 403–404 (1994). 7941228 10.1177/030098589403100401

[38] Katz, S. Coagulase Test Protocol. *Coagulase Test Protocol* (2010). https://asm.org:443/Protocols/Coagulase-Test-Protocol

[39] Cheng, A. G. et al. Contribution of coagulases towards Staphylococcus aureus disease and protective immunity. *PLOS Pathog*. **6**, e1001036 (2010). 20700445 10.1371/journal.ppat.1001036 PMC2916881

[40] Heilmann, C. Adhesion mechanisms of Staphylococci. *Adv. Exp. Med. Biol.* **715**, 105–123 (2011). 21557060 10.1007/978-94-007-0940-9_7

[41] Peetermans, M., Verhamme, P. & Vanassche, T. Coagulase activity by Staphylococcus aureus: A potential target for therapy?? *Semin Thromb. Hemost.* **41**, 433–444 (2015). 25973589 10.1055/s-0035-1549849

[42] Stepanović, S. et al. Quantification of biofilm in microtiter plates: overview of testing conditions and practical recommendations for assessment of biofilm production by Staphylococci. *APMIS* **115**, 891–899 (2007). 17696944 10.1111/j.1600-0463.2007.apm_630.x

[43] Singh, A., Walker, M., Rousseau, J. & Weese, J. S. Characterization of the biofilm forming ability of Staphylococcus pseudintermediusfrom dogs. *BMC Vet. Res.* **9**, 93 (2013). 23641755 10.1186/1746-6148-9-93 PMC3681638

[44] Marek, A. et al. Biofilm-Formation ability and the presence of adhesion genes in Coagulase-Negative Staphylococci isolates from chicken broilers. *Animals* **11**, 728 (2021). 33800098 10.3390/ani11030728 PMC7999041

[45] Stipkovits, L. & Kempf, I. Mycoplasmoses in poultry. *Rev. Sci. Tech. Int. Off Epizoot*. **15**, 1495–1525 (1996). 10.20506/rst.15.4.986 9190023

[46] Mugunthan, S. P., Kannan, G., Chandra, H. M., Paital, B. & Infection Transmission, pathogenesis and vaccine development against Mycoplasma gallisepticum. *Vaccines* **11**, 469 (2023). 36851345 10.3390/vaccines11020469 PMC9967393

[47] Jones, R. C. Avian reovirus infections. *Rev. Sci. Tech. Int. Off Epizoot*. **19**, 614–625 (2000). 10.20506/rst.19.2.1237 10935283

[48] Madhaiyan, M., Wirth, J. S. & Saravanan, V. S. Phylogenomic analyses of the Staphylococcaceae family suggest the reclassification of five species within the genus Staphylococcus as heterotypic synonyms, the promotion of five subspecies to novel species, the taxonomic reassignment of five Staphylococcus species to mammaliicoccus gen. Nov., and the formal assignment of Nosocomiicoccus to the family Staphylococcaceae. *Int. J. Syst. Evol. Microbiol.* **70**, 5926–5936 (2020). 33052802 10.1099/ijsem.0.004498

[49] Becker, K., Heilmann, C. & Peters, G. Coagulase-Negative Staphylococci. *Clin. Microbiol. Rev.* **27**, 870–926 (2014). 25278577 10.1128/CMR.00109-13 PMC4187637

[50] Brash, M. L. et al. Staphylococcosis. In *Avian Diseases Manual* (eds Boulianne, M. et al.) 134–136 (American Association of Avian Pathologists, 2013).

[51] Andreasen, C. B. Staphylococcosis. In *Diseases of Poultry, 14th Edition* (ed. Swayne, D. E.) 995–1003 (John Wiley &amp; Sons, Ltd, 2020).

[52] Wieliczko, A., Król, J., Piasecki, T., Mazurkiewicz, M. & Staroniewicz, Z. Występowanie i Charakterystyka Gronkowców Wyizolowanych Od Drobiu. *Med. Weter*. **58**, 348–352 (2002).

[53] Nawaz, M. S. et al. Biochemical and molecular characterization of erthromycin-resistant avian Staphylococcus spp. Isolated from chickens. *Poult. Sci.* **78**, 1191–1197 (1999). 10472846 10.1093/ps/78.8.1191

[54] Aarestrup, F. M. et al. Antimicrobial susceptibility and presence of resistance genes in Staphylococci from poultry. *Vet. Microbiol.* **74**, 353–364 (2000). 10831857 10.1016/s0378-1135(00)00197-8

[55] Marek, A. et al. Occurrence and characterization of Staphylococcus bacteria isolated from poultry in Western Poland. *Berl Münch Tierärztl Wochenschr*. 147–152. 10.2376/0005-9366-129-147 (2016). 27169153

[56] Milanov, D. et al. Slime production and biofilm forming ability by Staphylococcus aureus bovine mastitis isolates. *Acta Vet. (Beogr)* **60**, 217–226 (2010).

[57] Srednik, M. E. et al. Biofilm formation and antimicrobial resistance genes of coagulase-negative Staphylococci isolated from cows with mastitis in Argentina. *FEMS Microbiol. Lett.* **364**, fnx001 (2017). 10.1093/femsle/fnx001 28087612

[58] Fredheim, E. G. A. et al. Biofilm formation by Staphylococcus haemolyticus. *J. Clin. Microbiol.* **47**, 1172–1180 (2009). 19144798 10.1128/JCM.01891-08 PMC2668337

[59] Carneiro, C. R. W., Postol, E., Nomizo, R., Reis, L. F. L. & Brentani, R. R. Identification of enolase as a laminin-binding protein on the surface of S*taphylococcus aureus*. *Microbes Infect.* **6**, 604–608 (2004). 15158195 10.1016/j.micinf.2004.02.003

[60] Tristan, A. et al. Use of multiplex PCR to identify Staphylococcus aureus adhesins involved in human hematogenous infections. *J. Clin. Microbiol.* **41**, 4465–4467 (2003). 12958296 10.1128/JCM.41.9.4465-4467.2003 PMC193818

[61] Nemati, M., Hermans, K., Devriese, L. A., Maes, D. & Haesebrouck, F. Screening of genes encoding adhesion factors and biofilm formation in Staphylococcus aureus isolates from poultry. *Avian Pathol.* **38**, 513–517 (2009). 19937541 10.1080/03079450903349212

[62] Durairaj, V., Okimoto, R., Rasaputra, K., Clark, F. D. & Rath, N. C. Histopathology and serum clinical chemistry evaluation of broilers with femoral head separation disorder. *Avian Dis.* **53**, 21–25 (2009). 19431999 10.1637/8367-051908-Reg.1

[63] Zhang, M., Shi, C. Y., Zhou, Z. L. & Hou, J. F. Bone characteristics, histopathology, and chondrocyte apoptosis in femoral head necrosis induced by glucocorticoid in broilers. *Poult. Sci.* **96**, 1609–1614 (2017). 28339757 10.3382/ps/pew466

